# Editorial: Microbe decoration and biofabrication for drug delivery

**DOI:** 10.3389/fbioe.2024.1415129

**Published:** 2024-04-30

**Authors:** Weiliang Hou, Zunzhen Ming, Sisi Li, Xue Li, Feng Wu

**Affiliations:** ^1^ Tenth People’s Hospital Affiliated to Tongji University, Shanghai, China; ^2^ Shanghai Cancer Institute, Renji Hospital School of Medicine, Shanghai Jiao Tong University, Shanghai, China; ^3^ Department of Biological Sciences, National University of Singapore, Singapore, Singapore

**Keywords:** bacteria, drug delivery, cancer therapy, nanocoating, immunoregulation

Microbes take part in various physiological metabolic processes of our bodies, involving digestion, nutrition, metabolism, and immunity, which are closely related to the occurrence and development of various intestinal and extra-intestinal diseases. Recent studies have witnessed the dramatic developments of bacteria and their derivatives mediated biotherapies and biotic/abiotic hybrid therapies. However, surface-associated immunogenicity and dose-dependent virulence and limited efficacy restrict the further development and clinical translation of bacteria-based therapies. To tackle the above issues, various surface decoration strategies derived from physical, chemical, and biological means have been proposed for microbes and their derivatives, which realize the high-efficient combination between bacteria and diverse materials, such as contrast agents, anticancer drugs, nanoparticles and polymers. These organic/inorganic or biotic/abiotic hybrid systems circumvent the inadequacies and amplify the advantages of bacteria-based biotherapies, which will gain satisfactory outcomes in the diagnosis and treatment of diseases.

Liu et al. designed a facile and efficient encapsulation of single cells relying on the massive and controllable production of droplets and collagen-alginate microgels using a microfluidic device. High monodispersity and geometric homogeneity of both droplet and microgel generation were experimentally demonstrated based on the well-investigated microfluidic fabricating procedure. The reliability of the microfluidic platform for controllable, highthroughput, and improved single-cell encapsulation in monodisperse droplets and microgels was also confirmed. A single-cell encapsulation rate of up to 33.6% was achieved based on the established microfluidic operation. The introduction of stromal material in droplets/microgels for encapsulation provided single cells an *in vivo* simulated microenvironment. The single-cell operation achievement offers a methodological approach for developing simple and miniaturized devices to perform single-cell manipulation and analysis in a high-throughput and microenvironment-biomimetic manner.

Almuhayawi et al. used drop collapse, emulsification activity, and oil displacement assays to screen Halophilic bacteria from the Red Sea solar saltern in Egypt for producing biosurfactants and emulsifiers. Halobacterium jilantaiense strain JBS1 was the most effective strain of the Halobacteriaceae family. It had the best oil displacement test and emulsification activity against kerosene and crude oil, respectively. Among the ten isolates, it produced the most promising biosurfactant, also recognized by the GC-MASS library. This study evaluated biosurfactants from halophilic bacteria as potential antiviral drugs. Some of the computer methods we use are molecular docking, ADMET, and molecular dynamics. Molecular docking and molecular dynamics make the best complexes with 5VZ6 HIV-RT and flavone (C25) and 5wz3 ZV-RdRP and ethyl cholate (C8) Testing for ADMET toxicity on the complex revealed that it is the safest medicine conceivable. The 5VZ6-C25 and 5wz3-C8 complexes also followed the Lipinski rule. Finally, extreme settings require particular adaptations for stability, and extremophile biosurfactants may be more stable.

Bacterial cellulose (BC) is generated by certain species of bacteria and comprises polysaccharides with unique physical, chemical, and mechanical characteristics. To extend its applications in drug delivery, modifications of native bacterial cellulose are widely used to improve its properties. Liang al. presented a brief introduction to bacterial cellulose and its production and fabrication, followed by up-to-date and in-depth discussions of modification.

The use of live bacteria, engineered bacteria, or bacterial derivatives to deliver antitumor drugs to specific tumor sites for controlled release has emerged as a promising therapeutic tool. Ongoing research in this field holds great potential for further developing more efficient and personalized cancer therapies, such as *E. coli*, *Salmonella*, *Listeria*, and bacterial derivatives like outer membrane vesicles (OMVs), which can serve as vehicles for drugs, therapeutic proteins, or antigens. Song et al. describe the advances, challenges, and future directions of research on using live bacteria or OMVs as carriers or components derived from bacteria of delivery systems for cancer therapy.

The recently developed nanotechnology offers a new strategy to address this problem by developing drug-carrying nanoparticles with enhanced water solubility and targeting capacity, prolonged duration, and reduced side effects. Du et al. firstly discussed the pathogenesis of Abdominal aortic aneurysm (AAA), the methods of diagnosis and treatment that have been applied clinically, followed by the review of research progressions of constructing different drug-loaded nanoparticles for AAA treatment using engineered nanoparticles. In addition, the feasibility of extracellular vesicles (EVs) and EVs based nanotechnology for AAA treatment in recent years are highlighted, together with the future perspective.

This Research Topic was to provide comprehensive coverage of the relevant topics concerning the new strategies and materials of microbe decoration for the diagnosis and treatment of diseases. Statistically, a total of 5 manuscripts consisting of 2 original articles and 3 review papers were published in the Special Research Topic within 11 months. Despite that bacteria and their derivatives mediated biotherapies for drug delivery is in the infancy stage, we believe explosive development will come with the progress of nanotechnology and biotechnology.

